# Goal attainment scaling in the heart failure and stroke resilience caregiver intervention pilot study

**DOI:** 10.1186/s41687-026-01082-5

**Published:** 2026-05-16

**Authors:** Catherine A. Clair, Samantha N. Curriero, Martha Abshire Saylor

**Affiliations:** 1 Johns Hopkins Bloomberg School of Public Health, 2213 McElderry St, 2nd Floor, Baltimore, MD 21205 USA; 2 Johns Hopkins School of Nursing, Baltimore, MD USA

**Keywords:** Caregiver, Goal attainment scaling, Heart failure, Stroke, Goal achievement

## Abstract

**Background:**

Caregivers often serve as proxies for patients or inform patient-caregiver dyadic findings in interventions. We conducted a randomized waitlist control pilot of a caregiver support program focused on caregivers (not dyads): Heart Failure and Stroke Resilience Intervention for Caregivers (HEROIC). Goal attainment scaling (GAS) was used to measure goal progress and achievement; this measure is person-centered but has not been used to test prior caregiving interventions.

**Methodology:**

HEROIC was tested in a pilot study. Participants were randomized to immediate intervention or waitlist control groups. GAS data was collected at baseline, 12 weeks, and 24 weeks and a final interview to assess feasibility and acceptability was conducted at 24 weeks. Raw GAS data was converted to t-scores. We compared goal achievement for the overall sample, by group assignment (immediate intervention vs. waitlist control), and by goal domain. Two questions in the final interview focused on acceptability and feasibility of GAS were summarized.

**Results:**

Fifty caregivers were consented, and 31 completed baseline data collection. Caregivers (*n* = 31) were on average 58 years old (SD: 11), 84% identified as female and 45% identified as Black. Twenty-six participants completed GAS at 12 weeks, and 19 participants completed GAS at 24 weeks. Twenty-one (80.8%) and 16 (84.2%) caregivers met or exceeded their goal at 12 weeks and 24 weeks respectively. For the immediate intervention group, the mean change in scores pre-post intervention was 19.1 (SD: 9.4), ranging from 0 to 30. For the waitlist control group, the mean change in scores pre-post intervention was 13.8 (SD: 10.6), ranging from 0 to 30. For the immediate intervention group during their maintenance period (12 to 24 weeks), the mean change in scores was 20.9 (SD: 11.4), ranging from 0 to 30. All 19 caregivers were interviewed at 24 weeks and reported that GAS was simple to complete and reported satisfaction with the activity.

**Conclusions:**

For clinical trials, GAS may serve as a key outcome and provide rich effectiveness data. While GAS represents investment of time and resources, the approach is valuable. We recommend future research to inform streamlining GAS while maintaining its person-centeredness.

**Trial registration:**

ClinicalTrials.gov, NCT03963583, Registered 24 May 2019, https://clinicaltrials.gov/study/NCT03963583?term=Heart%25;20Failure%25;20Resilience%25;20Intervention%25;20for%25;20Caregivers%25;20(HEROIC)%26;rank=1.

**Supplementary Information:**

The online version contains supplementary material available at 10.1186/s41687-026-01082-5.

## Background

Goal setting and achievement is a common outcome measure in social and behavioral support interventions, particularly to measure individual (patient) and/or interpersonal (patient and caregiver) response [13]. However, the literature has identified several issues with measuring progress toward goals in interventions. A recent scoping review by Crawford and colleagues highlighted barriers at the individual, interpersonal, and organizational levels [12]. Some of these barriers include lacking standard approach, differing perceptions between the patient (goal setter) and provider perspective, and varying forms of clinical documentation [12]. Additionally, Baker and colleagues identified specific challenges with goal setting in the rehabilitation context; clinicians needed consistent processes but desired some level of flexibility [5]. One method of objectively measuring individualized goal progress and achievement is goal attainment scaling.

Goal attainment scaling (GAS) is a patient-centered method that incorporates qualitative and quantitative information to measure goal achievement [7, 14, 15, 17, 19]. Initially used in mental health settings [17], GAS has been adapted for use in settings such as rehabilitation [19] and drug trials [14] as well as populations, ranging from youth [9, 30] to older adults [7, 11, 15]. GAS literature has engaged caregivers in goal setting and goal rating for patient populations [10]. However, to our knowledge, GAS has not been investigated as an outcome for a caregiver-focused intervention study.

Caregivers of persons living with cardio- and cerebrovascular disease are often balancing significant caregiving tasks with their own needs and quality of life [26, 34]. Due to the nature of managing these conditions, commonly with a lack of formalized training, caregivers often experience high levels of stress [4, 35]. To address caregivers’ needs and provide support, we conducted a randomized waitlist control pilot of a caregiver support program: Heart Failure and Stroke Resilience Intervention for Caregivers (HEROIC). Caregivers served as the sole participants (rather than as a member of patient-caregiver dyad) and set goals for themselves. GAS was used to measure goal progress and achievement throughout the study [22]. We completed a prior pilot of HEROIC with heart failure caregivers alone [3, 29] and examined goal-setting [1]. Caregivers set multiple action plans (*N* = 85) and achieved approximately 54% (*n* = 46) [1]; however, the study did not use an objective measure of goal achievement. In this study, we made minor adaptations of the intervention to include stroke caregivers and added GAS as a measure of goal progress and achievement. We have two objectives for this analysis. First, we aim to assess goal achievement using GAS at three timepoints over the study period. Second, we investigate the feasibility and acceptability of conducting GAS within the context of the HEROIC intervention study. Our reporting is guided by best practices outlined in a systematic review by Cheema and colleagues [10].

## Methods

### Study design

We conducted a randomized waitlist-controlled pilot study to examine feasibility, acceptability and preliminary effectiveness for caregivers of persons living with heart failure and/or stroke. This study was approved by the Johns Hopkins Medicine Institutional Review Board (IRB00277814).

Requirements for inclusion included the following: (1) the patient had experienced decompensated heart failure or stroke in the last 6 months (i.e., hospitalization); (2) the caregiver lived with the patient or visited them to provide care more than 3 times per week; and (3) the caregiver was 18 years or older. Caregivers were excluded from the study if they themselves had a terminal diagnosis or were cognitively impaired (as determined by 6-item screener) [27]. We employed a multi-method recruitment strategy, which included (1) provider referral; (2) flyer distribution in cardiology and neurology clinics; and (3) outreach to participants enrolled in other Johns Hopkins heart failure-related studies. Once contact was made with the caregiver, they were screened for eligibility. If the caregiver was eligible and interested in participating, then electronic consent was obtained (by phone). As a pilot study, sample size was determined for feasibility and acceptability; we aimed for enrollment of 36 caregivers and anticipated an attrition rate of 30% [33].

The HEROIC program consists of 5 nurse-led, virtual sessions over a 10-week period. The program includes the following activities: (1) Whole Person Assessment; (2) Life Purpose Statement; (3) Social Support Circles; (4) Instrumental Support Resource Identification; and (5) Action Planning (i.e., self-care, physical activity). HEROIC was iterated from the Caregiver Support intervention, and expanded description of the intervention components is published elsewhere [3].

Following baseline data collection, caregivers were provided with their intervention materials and randomized to either immediate intervention or waitlist control groups. The immediate intervention group received the HEROIC intervention between baseline (0 weeks) and 12 weeks, while the waitlist control group received the intervention between 12 weeks and 24 weeks. Data collection occurred for all caregivers at baseline, 12 weeks, and 24 weeks.

### Goal attainment scaling personnel training

A single data collector (SNC) blinded to group assignment conducted all GAS data collections in the study. Training of the data collector included a mix of didactic methods and role-play. The trainers included CAC, who has previous training and research experience using GAS, and MAS, who provided expertise in heart failure care and caregiver support. The first training was 1 h and involved explanation of GAS and review of the data collection worksheet and instruments. The subsequent trainings (cumulative total of 3 h) involved role-play between the data collector and MAS, with CAC providing feedback on whether the scaling of goals was precisely described at each level. Throughout the study, the data collector met with CAC and MAS to seek feedback and ensure appropriate documentation. Trained lay interviewers have been found to be acceptable facilitators of GAS in research and clinical encounters [31].

### Data collection and analysis

Goal attainment scaling data was collected at baseline, 12 weeks, and 24 weeks. Initial baseline data collections occurred in-person. To assess feasibility of remote GAS data collection, the majority of the baseline and all 12- and 24-week data collections occurred by phone, which has been demonstrated as feasible in older adult populations [31]. Additional survey data was collected at the three timepoints; however, participants were blinded to their other test scores.

At baseline data collection, the participant was asked to select a goal domain: (a) In the next 12 weeks, I will take care of my own health and well-being or (b) In the next 12 weeks, I will prioritize caregiving and getting the support I need to help my loved one. Following selection of a domain, the participant was guided in completing the 5-point GAS. This goal inventory included examples of how the goal domains could be scored in the 5-point GAS. Participants were encouraged to complete all 5 levels of the scale. All goals were set at a baseline of -1 (“Where are you now?”) with an intended achievement of 0 (“Where do you want to be?”) [15]. The determination of -1 and 0 were caregiver specified. For example, “walk 1 mile per day” may represent one caregiver’s baseline (-1) and another caregivers expected achievement (0). The participant also rated the difficulty and confidence in achieving the goal on a 5-item Likert scale (Not at all, A little, Somewhat, Quite a bit, Very). Only one goal was set during each data collection session. The GAS worksheet used with HEROIC participants is included as Supplementary Materials.

At 12 weeks, the participant was asked to narratively describe their progress toward goal achievement and to score their progress using the 5 levels set at baseline. Finally, participants were asked about their next steps. They had the opportunity to “Continue to work on the same goal and do not revise scaling,” “Continue to work on the same goal and revise the scaling,” or “Work on a new goal.” If participants opted to revise the scaling, they had the opportunity to revise the outcomes in the 5-point GAS. If participants opted to work on a new goal, they repeated the process from baseline by selecting a goal domain and defining outcomes related to achievement their new goal. Following completion of 12-week scoring, baseline scores were reset to t-score = 40 [15].

At 24 weeks, participants were again asked to narratively describe their progress on their goal from the 12-week data collection and to score their progress using the 5 outcomes. Over the course of the study, each caregiver set two goals: one at baseline and one at 12 weeks. Additionally, they completed a final interview to assess feasibility and acceptability of the HEROIC intervention.

Following completion of the study, raw GAS data was converted to a t-score. Baseline t-scores were set to 40, and all scores were unweighted. Goal achievement was defined as a t-score of 50 or greater at follow-up data collection timepoints. We compared goal achievement for overall sample, by group assignment (immediate intervention vs. waitlist control), and by goal domain (A vs. B). Finally, two questions of the final interview were focused on acceptability and feasibility of GAS. Responses to these questions were reviewed and summarized by CAC and MAS using basic content analysis to assess participants’ feedback.

## Results

### Sample

**Fig. 1:**
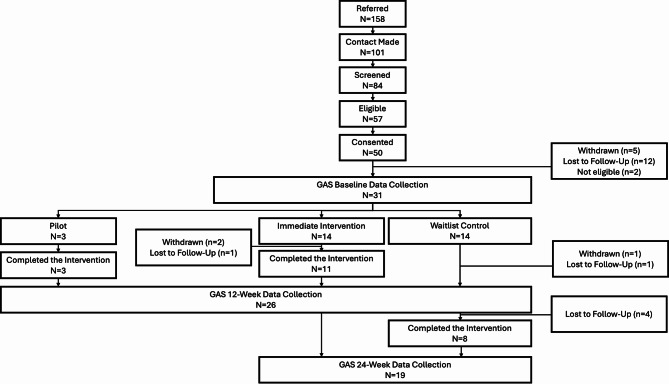
CONSORT flow diagram for HEROIC

Fifty caregivers were consented, and 31 completed baseline data collection. The first three caregivers were included as open-label pilot participants and were not randomized. The remaining 28 caregivers were randomized into the immediate intervention (*n* = 14) and waitlist control (*n* = 14) following baseline data collection. Figure 1 includes a CONSORT flow diagram. Following consent, eight caregivers withdrew from participation. Reasons included feeling overwhelmed (*n* = 6), family conflict (*n* = 1), and bereavement (*n* = 1). The one bereaved caregiver was offered and declined to participate in a bereavement-version of the HEROIC program.

Caregivers (*n* = 31) were on average 58 years old (SD: 11), 84% identified as female and 45% identified as Black. They supported patients living with moderate to severe functional impairment and 5.5 ± 2.3 instrumental activities of daily living (IADL) on average. Over half of caregivers reported financial strain (52%), which was defined as “Having just enough money” or “Having not enough money” to make ends meet by the end of the month. Most of the caregivers in the study supported persons with heart failure (*n* = 19) and 12 caregivers supported persons living with stroke. All three open-label pilot participants were caregiving for persons with heart failure. All sociodemographic characteristics are included in Table 1.

**Table 1:** Sociodemographic characteristics of caregivers in HEROIC

Variable	Total (*N* = 31) *N* (%)
Age (mean ± SD)	58.4 (11.4)
Gender	
Male	5 (16.1)
Female	26 (83.9)
Race	
White	17 (54.8)
Black	14 (45.2)
Employment Status	
Working now	13 (41.9)
Retired	6 (19.4)
Disabled/sick leave	4 (12.9)
Looking for work/Homemaker/Student/Other	8 (3.2)
Experiencing Financial Strain	16 (51.6)
Caregiver Type	
Heart failure	19 (61.3)
Stroke	12 (38.7)
Relationship to Care Recipient	
Spouse or partner	18 (58.1)
Parent	1 (3.2)
Child	9 (29.0)
Other family	2 (6.5)
Friend	1 (3.2)
Functional Dependence	
Heart failure: Assistance with ≥ 1 Activity of Daily Living	4 (21.1)*
Stroke: Barthel Index of ≤ 90	6 (50.0)
Instrumental Activities of Daily Living (IADL) Dependence	
Total sample assisting with > 2 IADLs	27 (87)
Mean ± SD across total sample	5.5 (2.3)

*Missing data for 2 heart failure caregivers

### Goal attainment scaling and achievement

Twenty-six participants completed data collection at 12 weeks, and 19 participants completed data collection at 24 weeks. Attrition was due to loss to follow-up and withdrawal from the overall study; no participant actively enrolled refused to complete scaling. The three open-label pilot participants did not complete GAS data collection at 24 weeks as per the study protocol.

**Fig. 2:**
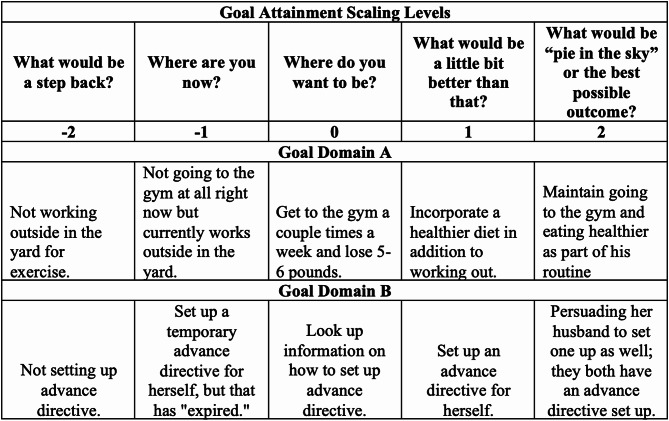
Example of 5-level goal attainment scaling for goal domains A and B

At baseline, 18 participants selected goal domain A (“In the next 12 weeks, I will take care of my own health and well-being”) and 13 selected goal domain B (“In the next 12 weeks, I will prioritize caregiving and getting the support I need to help my loved one”). The majority of heart failure caregivers (68.4%) selected goal domain A, while the majority of stroke caregivers (58.3%) selected goal domain B. All 5 levels of GAS were specified at baseline and 12 weeks for all caregivers. Examples of scaling for goal domains A and B are included in Fig. 2.

**Fig. 3:**
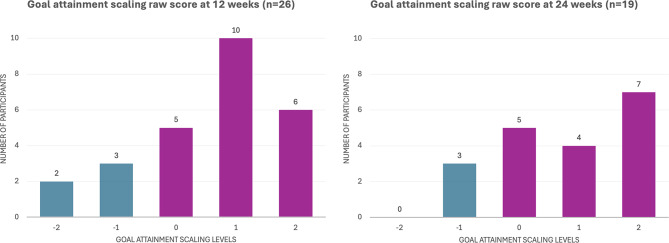
Goal attainment scaling raw score for overall sample

Twenty-one (80.8%) and 16 (84.2%) caregivers met or exceeded their goal at 12 weeks and 24 weeks respectively (see Fig. 3).

**Fig. 4:**
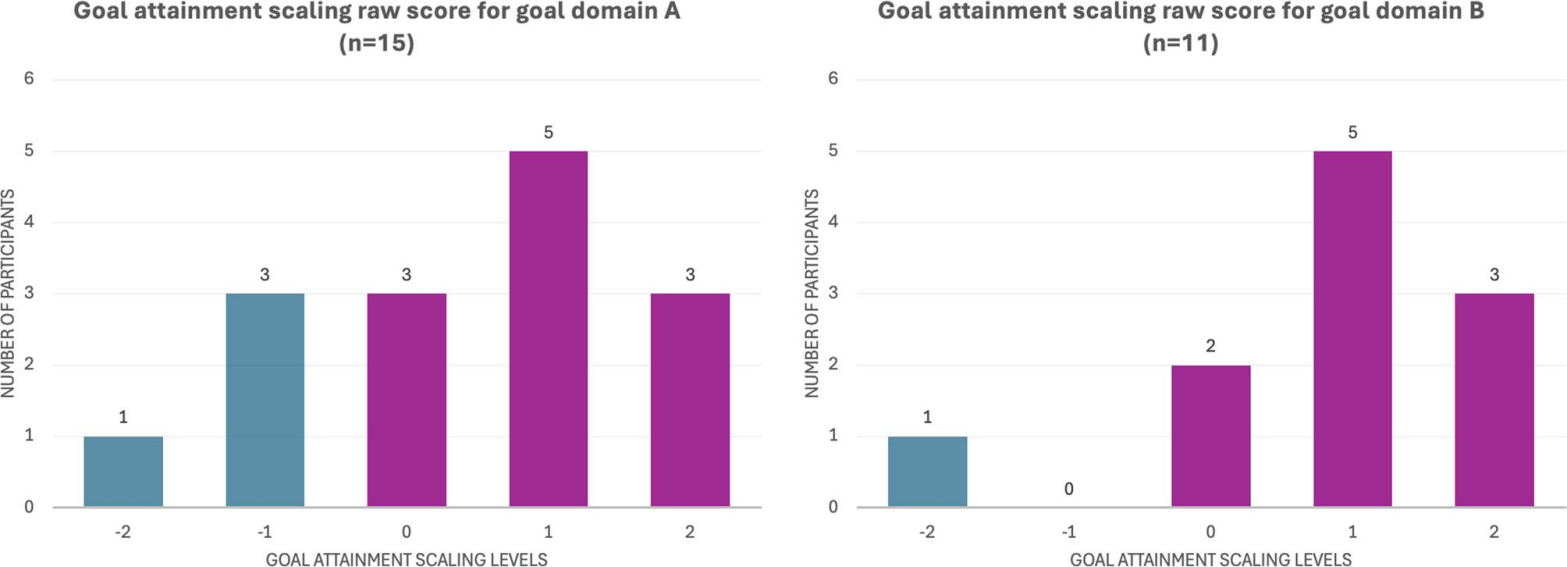
Goal attainment scaling raw score at 12 weeks by goal domain

At 12 weeks, 11 (73.3%) caregivers who scaled using goal domain A met or exceeded their goal compared to 10 (90.1%) caregivers who scaled using goal domain B (see Fig. 4).

**Fig. 5:**
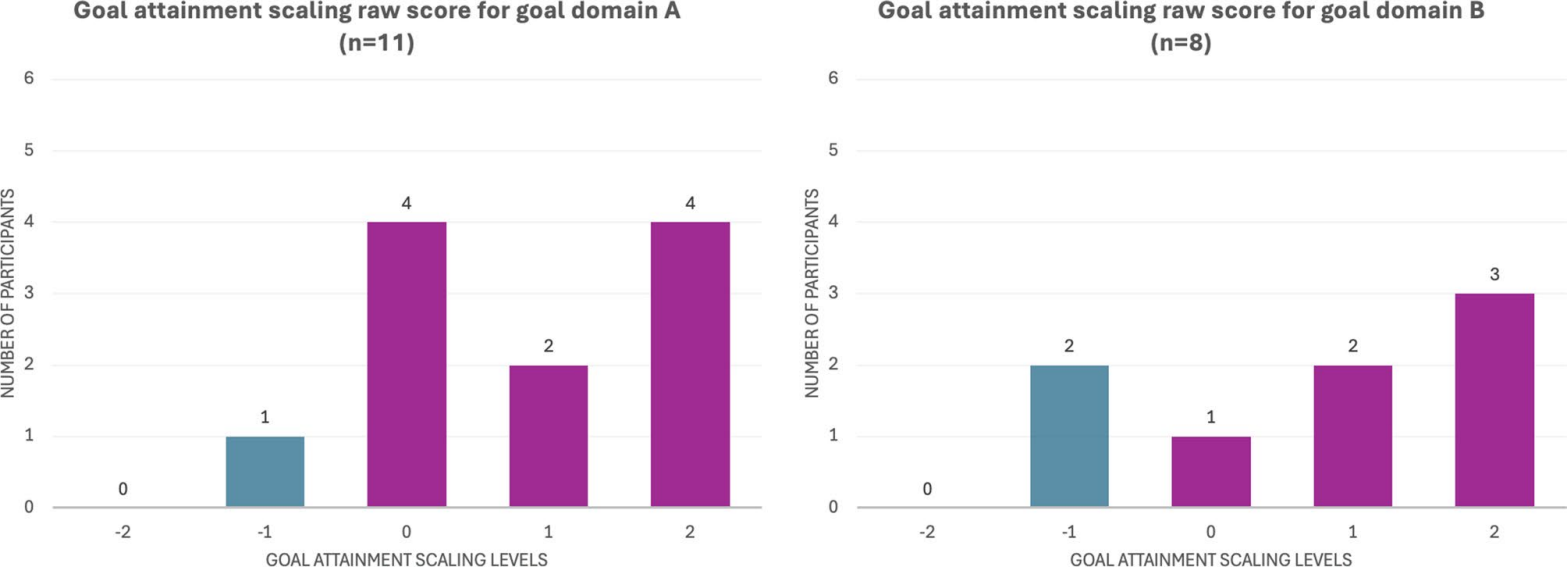
Goal attainment scaling raw score at 24 weeks by goal domain

Similarly, at 24-weeks, 10 (90.9%) caregivers who scaled using goal domain A met or exceeded their goal compared to 6 (75.0%) caregivers scaled using goal domain B (see Fig. 5).

For the immediate intervention group, the mean change in scores pre-post intervention was 19.1 (SD: 9.4), ranging from 0 to 30. During their wait period, for the waitlist control group, the mean change in scores from baseline to 12 weeks was 12.5 (SD: 14.2), ranging from − 10 to 30. For waitlist control group, the mean change in scores pre-post intervention was 13.8 (SD: 10.6), ranging from 0 to 30. During their maintenance period, for the immediate intervention group, the mean change in scores from 12 weeks to 24 weeks was 20.9 (SD: 11.4), ranging from 0 to 30 (see Table 2). When comparing the first 12 weeks, in which the intervention group received intervention and the waitlist control group did not, we saw a mean difference of 6.6 (SD: 5.1) in scores between the groups, though this difference was not statistically significant. After scoring achievement at 12 weeks, baseline score was reset to t-score = 40. When comparing the second 12 weeks, in which the intervention group was in maintenance phase and the waitlist control received the intervention, the mean difference in scores widened (Mean difference: 7.2, SD: 5.1) and was not statistically significant.

**Table 2:** Goal attainment scaling t-score at three timepoints overall and by group assignment

Mean (SD)	Overall sample (*N* = 31)	Group Assignment			Group Comparison	
		Open-Label Pilot (*n* = 3)	Immediate intervention (*n* = 14)	Waitlist control (*n* = 14)	Mean difference	Two-sample Wilcoxon rank-sum (Mann-Whitney) test
Baseline t-score	40 (0)	40 (0)	40 (0)	40 (0)	-	-
		Received intervention				
		*n* = 3	*n* = 14	*n* = 12		
12-week t-score	54.6 (12.1)	56.7 (11.5)	59.1 (9.4)	52.5 (14.2)	6.6 (5.1)	*p* = 0.3092
Baseline (reset) at 12 weeks	40 (0)	-	40 (0)	40 (0)		
				Received intervention		
		-	*n* = 11	*n* = 8		
24-week t-score	57.9 (11.3)	-	60.9 (11.4)	53.8 (10.6)	7.2 (5.1)	*p* = 0.1602

Additionally, the majority of participants chose to continue with their goal set at baseline during the entire study period. At 12-week data collection, 15 participants chose to “Continue to work on the same goal and not revise scaling,” four participants chose to “Continue to work on the same goal and revise the scaling,” and seven chose to “Work on a new goal.”

### Feasibility and acceptability

All nineteen caregivers who completed the final data collection were interviewed at study completion. The data collector asked the initial question, “How were the data collection visits?” with a follow-up prompt regarding time (“Were they a reasonable amount of time?”). Thirteen caregivers responded positively to GAS and six provided no comment; no caregivers responded negatively. Overall, caregivers used words like “good,” “delightful,” “beneficial,” “great,” and “fine” to describe their experience with GAS. One caregiver noted that the experience was “better than expected.” In terms of time, caregivers noted that the time to complete the activity was “reasonable” and “not too much.” One caregiver felt that the activity could be shortened but reiterated that the timing should be tailored to the participant, their needs, and their schedule. Finally, caregivers provided feedback on working with the data collector, with one participant sharing that it was “easy to talk [with her].” Others described the data collector as “pleasant,” “open,” “friendly”, “forgiving,” and “terrific.”

## Discussion

As part of the HEROIC randomized waitlist control trial, we aimed to assess goal achievement using GAS and the feasibility and acceptability of using this method. We found that caregivers were able to set and evaluate their goals using GAS and that the method was feasible and acceptable. Overall, this structured goal setting offered a strengths-based outcome measure to assess effectiveness of the HEROIC intervention while promoting efficiency for study operations. For clinical trials, particularly those implementing behavioral and social support interventions, GAS may serve as a key outcome and provide richer effectiveness data compared to survey measures alone, giving insight to how change in survey measures was achieved. As part of our data collection, caregivers provided narrative progress in addition to their GAS score. Although that data is not presented in this analysis, we feel that it added additional context to the GAS score, particularly for caregivers managing acute crises with their loved ones. We recommend that studies using GAS, particularly if working with caregivers or other populations that experience acute crises or disruptions to schedule, also capture this data. Examples of narrative progress are included in Table 3.

**Table 3:** Examples of narrative progress compared to follow-up goal attainment scaling score

Participant Baseline Expected Level (0)	GAS Score at Follow-Up	Narrative Progress
Walk at least 3 times a week over the next two weeks.	-2	“It’s a very unusual experience for me because I never foresaw what was coming…I [walked] until Christmas, then my son ended up with a psychotic break and landed in the ER. My schedule got shot to pieces because I had to be on call all hours of the day. [I am] only walking sporadically.” -- Participant 1
Eat more fiber and veggies.	-1	“Not good. [It] started out good, just a lot of social events. [I did] not eat the cookies like I normally [would].” -- Participant 2
Get [patient] involved in a stroke support group.	0	“I did the majority of it. He did go to support group and will continue to go to the support group.” -- Participant 3
Having more resources available.	1	“I’ve been taking care of myself, going to physical therapy, started walking, some more exercise, some more crocheting. Regarding the handyman, I’ve been doing the projects on my own and I am accomplishing them…it makes me feel really good.” -- Participant 4
See the orthopedist and keep the appointment.	2	“100% accomplished. I saw the orthopedist and walked down to the beach from coastal highway.” -- Participant 5

Additionally, as it pertains to clinical trial study design, our analysis highlights the importance of a “maintenance” timepoint, which was the third data collection for participants in the immediate intervention group. Many interventions focus on pre-post reporting, which leaves the question of an intervention’s continued – and potentially lasting – effect. We found that the immediate intervention group had a higher average t-score at 24-weeks compared to 12-weeks, suggesting that the activities of HEROIC promoted goal progress and achievement, even after active intervention participation. Surprisingly, when comparing mean difference in score at 24-weeks, the gap between groups widened: The waitlist control group saw little difference from their scores at the end of their waiting period in comparison to their scores post-intervention. This may speak to the structure of the 6-month study timeline per participant allowing for goals to become habits. While goals are explicitly set, they directly inform habit formation (implicit). Habits emerge from the gradual learning of associations between responses and, once a habit is formed, a mediating goal becomes unnecessary to trigger a response [21, 36]. Wood and Neal described the cyclical nature of goals and habits: Goals direct habits, habits and goals interact, and habits inform goals [36]. For caregivers, it may be challenging to set goals due to the lack of time, internal and external resources and support. Lengthier programming, like HEROIC, may offer the opportunity for self-prioritization and allow sufficient time for goals to interact with and form healthier habits.

Given that participants were randomized to study group and not randomized by goal domain, it is challenging to speak to the differences in achievement by goal domain. Our results indicated that more caregivers selecting goal domain A (“In the next 12 weeks, I will take care of my own health and well-being”) achieved their goals compared to those who selected goal domain B (“In the next 12 weeks, I will prioritize caregiving and getting the support I need to help my loved one”). The ability to select one domain over the other may be influenced by factors such as readiness [6, 32], patient condition severity [16], and experience of caregiver burden [23]. This literature is primarily focused on dementia and cancer caregivers, and little has been explored within the context of heart failure and/or stroke caregiving. These factors, particularly patient condition severity and trajectory (i.e., heart failure, stroke), are likely to influence goal achievement generally and within the domains. While some may propose randomizing by goal domain, we would discourage this practice. Allowing caregivers to select their own goals is person-centered and more likely to align with the circumstances of caregiving [2]. However, adding questions into feasibility and acceptability interviews post-study completion that inquire into the decision-making processes and selection of goal domain may offer additional insight.

There is debate that the benefits of GAS do not outweigh the challenges, such as cost and time efficiency [22, 24]. To counter this, some researchers have proposed shortening the scale to three levels [20] and utilizing pre-populated levels [8] to facilitate use in research and clinical practice. Also, GAS was built on the foundation of measuring progress based on directly observable changes [28]. With community-based or clinic-based implementation of GAS for caregivers, achievement is based upon self-report [22]. Broadening the utility of GAS for self-reported or non-observable change is important and requires further exploration; we encourage exploration of pragmatic modifications to GAS and its impact on validity and reliability. In HEROIC, GAS did require a part-time data collector, scheduling, and completing a data collection session with each participant for the three timepoints. However, similar to previous qualitative literature with older adults living with complex care needs [11], our participants felt that GAS added value to the HEROIC study. By creating goal domains related to the intervention targets, we guided caregivers in their decision-making while ultimately allowing them to specify the five levels as they saw fit. We believe there may be additional, innovative ways to use GAS efficiently in a clinical trial while maintaining its person-centeredness, worthy of further exploration.

### Strengths & limitations

Our analysis has several limitations. First, with the limited sample size, we were unable to compare goal achievement by heart failure and stroke caregivers. Given that these caregivers selected different goal domains (i.e., Goal Domain A vs. B), there may be diagnosis-specific caregiver needs regarding goal progress and achievement. Second, our feasibility and acceptability data only represent 19 caregivers. We do not have GAS data from the 9 randomized caregivers that withdrew and were lost to follow-up, which may limit our ability to conclude feasibility and acceptability for all caregivers in the study. It is unknown if GAS contributed to their attrition; however, based on reasons given by caregivers who withdrew, we believe it is unlikely to have contributed. Third, some caregiver goals were difficult to operationalize, as seen in Table 3 (e.g., “Eat more fiber and veggies”). We recognize the resulting pragmatic issue; our aim of the study was to center caregivers and goals, and for some caregivers, this represented their goals. Based on our study principles, we accepted this as a limitation. Fourth, we limited our participants to one goal. While there is promising literature on this approach [25], it may limit the responsiveness of GAS as an outcome measure and represent a potential loss of reliability [18]. Again, this represents a trade-off between psychometrics and implementation in a community-based setting. Finally, our data collector who conducted the GAS sessions also conducted the final interview (SNC), which may have resulted in social-desirability bias regarding the activity.

## Conclusion

We aimed to assess goal achievement in the HEROIC study using GAS at three timepoints (baseline, 12 weeks, and 24 weeks) and investigate the feasibility and acceptability of this approach. Overall, caregivers of persons living with heart failure and/or stroke responded positively to the method, and the majority were able to achieve their goals over the study period. While GAS may represent investment in terms of time and resources, the approach was valued by our participants. We recommend future research to inform streamlining GAS while maintaining its person-centeredness, as this will be critical for clinical practice and implementation.

## Supplementary Information

Below is the link to the electronic supplementary material.

Supplementary Material 1

## Data Availability

The dataset generated during and analyzed during the current study is not publicly available due the sensitive nature of data collected but is available from the corresponding author on reasonable request.

## Abbreviations

GAS: Goal attainment scaling
HEROIC: Heart Failure and Stroke Resilience Intervention for Caregivers
IADL: Instrumental activities of daily living
SD: Standard deviation
