# Supplementary material for: Goal attainment scaling in the heart failure and stroke resilience caregiver intervention pilot study
Source: J Patient Rep Outcomes. 2026 May 16;10:115. doi: 10.1186/s41687-026-01082-5 (PMC13357473; doi:10.1186/s41687-026-01082-5)
Supplement: Supplementary file 1 — Supplementary Material 1 [file 41687_2026_1082_MOESM1_ESM.docx]

The CONSORT-PRO Reporting Guidance Checklist

| Section/Topic | CONSORT-  PRO Item | Recommended Content | Page Addressed |
| --- | --- | --- | --- |
| **Title and Abstract** | | | |
|  | P1b | The PRO should be identified in the abstract as a primary or secondary outcome. | Title: Page 1; Abstract: Page 3 |
| **Introduction** | | | |
| Background and objectives | 2a | The scientific background and explanation of rationale of PRO assessment should be included. | Introduction: Pages 5-6 |
|  | P2b | The PRO hypothesis should be stated, and relevant domains identified, if applicable. | Not applicable. Pilot study focused on assessing feasibility and acceptability. |
| **Methods** | | | |
| Participants | 4a | PRO-specific criteria are required only if PROs were used for eligibility or stratification. | Not applicable. |
| Outcomes | P6a | Evidence of PRO instrument validity and reliability should be provided or cited if available including the person completing the PRO and methods of data collection (paper, telephone, electronic). | Methods: Page 8 |
| Sample size | 7a | Sample size determination is required only if PRO is a primary study outcome. | Not applicable. PRO was a secondary outcome, and pilot study was focused on feasibility and acceptability. |
| **Randomization** | | | |
| Statistical methods | P12a | Statistical approaches for dealing with missing data are explicitly stated. | Not applicable. No missing data from enrolled participants. |
| **Results** | | | |
| Participant flow | 13a | The number of PRO outcome data at baseline and at subsequent time points should be  transparent. | Figure 1 (CONSORT flow diagram) |
| Baseline data | 15 | PRO data in the table showing baseline demographic and clinical characteristics for each group should be included. | Table 1 |
| Numbers analyzed | 16 | For each group, the number of participants (denominator) included in each analysis and  whether the analysis was by original assigned groups) is required for PRO results. | Results: Pages 12-16; Figures 3-5; Table 2 |
| Outcomes and estimation | 17a | The estimated effect size and its precision such as 95% confidence interval should be presented for multidimensional PROs from each domain and time point. | Not applicable. |
| Ancillary analyses | 18 |  |  |
| **Discussion** | | | |
| Limitation | P20/21 | PRO-specific limitations and implications for generalizability and clinical practice should be  presented. | Discussion: Pages 18-19 |
| Interpretation | 22 | PRO data should be interpreted in relation to clinical outcomes including survival data, where relevant. | Not applicable. The population of interest is caregivers, and survival is a less relevant comparison. We did not collect clinical outcomes for caregivers as it was beyond the scope of this study. |

Calvert M, Blazeby J, Altman DG, et al. Reporting of patient-reported outcomes in randomized trials: the CONSORT PRO extension. *JAMA*. 2013;309(8):814-822. doi:10.1001/jama.2013.879

Note: The CONSORT-PRO Extension should be used with the CONSORT 2010 Statement and any other relevant CONSORT Extensions, found at consort-statement.org
